# Coping Well with Advanced Cancer: A Serial Qualitative Interview Study with Patients and Family Carers

**DOI:** 10.1371/journal.pone.0169071

**Published:** 2017-01-20

**Authors:** Catherine Walshe, Diane Roberts, Lynda Appleton, Lynn Calman, Paul Large, Mari Lloyd-Williams, Gunn Grande

**Affiliations:** 1 International Observatory on End of Life Care, Lancaster University, Bailrigg, Lancaster, United Kingdom; 2 The School of Nursing, Midwifery and Social Work, The University of Manchester, Manchester, United Kingdom; 3 Clatterbridge Cancer Centre NHS Foundation Trust, Wirral, United Kingdom; 4 Faculty of Health Sciences, University of Southampton, Southampton, United Kingdom; 5 User representative, Liverpool, United Kingdom; 6 Institute of Psychology, Health and Society, University of Liverpool, Liverpool, United Kingdom; University of Stirling, UNITED KINGDOM

## Abstract

**Objectives:**

To understand successful strategies used by people to cope well when living with advanced cancer; to explore how professionals can support effective coping strategies; to understand how to support development of effective coping strategies for patients and family carers.

**Design:**

Qualitative serial (4–12 week intervals) interview study with people with advanced cancer and their informal carers followed by focus groups. The iterative design had a novel focus on positive coping strategies. Interview analysis focused on patients and carers as individuals and pairs, exploring multiple dimensions of their coping experiences. Focus group analysis explored strategies for intervention development.

**Participants:**

26 people with advanced (stage 3–4) breast, prostate, lung or colorectal cancer, or in receipt of palliative care, and 24 paired nominated informal/family carers.

**Setting:**

Participants recruited through outpatient clinics at two tertiary cancer centres in Merseyside and Manchester, UK, between June 2012 and July 2013.

**Results:**

45 patient and 41 carer interviews were conducted plus 4 focus groups (16 participants). People with advanced cancer and their informal/family carers develop coping strategies which enable effective management of psychological wellbeing. People draw from pre-diagnosis coping strategies, but these develop through responding to the experience of living with advanced cancer. Strategies include being realistic, indulgence, support, and learning from others, which enabled participants to regain a sense of wellbeing after emotional challenge. Learning from peers emerged as particularly important in promoting psychological wellbeing through the development of effective ‘everyday’, non-clinical coping strategies.

**Conclusions:**

Our findings challenge current models of providing psychological support for those with advanced cancer which focus on professional intervention. It is important to recognise, enable and support peoples’ own resources and coping strategies. Peer support may have potential, and could be a patient-centred, cost effective way of managing the needs of a growing population of those living with advanced cancer.

## Introduction

Many different healthcare workers provide care to people with advanced cancer as the number of people with advanced cancer increases, and the likely trajectory of their disease course elongates[1]. Knowing how best to provide effective supportive care to someone with this diagnosis is therefore a core healthcare skill. Most approaches focus on professional interventions to ameliorate negative consequences of the diagnosis such as anxiety and depression[2]. Our work is thus novel in its focus on the identification of person centred positive coping strategies to enhance wellbeing that can be facilitated and developed beyond the clinical environment.

People experiencing advanced cancer can experience adverse psychological impacts from this diagnosis. The prevalence of interview-defined major depression is estimated at 15–19% in palliative and cancer care, with 10% experiencing anxiety, and 38% any mood disorder [3–5]. In the UK nearly 30, 000 people with cancer were in receipt of antidepressant medication for a mean of 12.2. weeks[4] despite questionable effectiveness in reducing depression scores[6] for those with advanced cancer. Current clinical guidance suggests that all healthcare professionals should be able to provide basic psychological support, but promotes a tiered approach where increasing professional expertise in psychological care should be sought with increasing severity or complexity of problems[2]. Access to such services can, however, be problematic due to availability issues[7], and many healthcare professionals are known to focus on physical rather than psychological symptoms[8, 9]. When psychological issues are identified this does indeed increase referrals to professional psychological support services[10]. However, the evidence on whether such psychological interventions reduce anxiety and depression is equivocal [11]. Psychological issues are therefore common in those with advanced cancer, but the evidence base for effective interventions addressing issues such as depression and anxiety is scarce.

A major conceptual issue with these approaches to care are that they largely ignore the coping strategies that people with advanced cancer and their informal carers use, and can pathologise psychological experience [12–21]. It is known that a range of appraisal and coping strategies are associated with lower incidence of psychological responses such as stress and distress [22–27]. However, knowing about the association between coping strategies and psychological response does not enable clinicians to understand how people operationalise these coping strategies or how to support their development. In addition, much of this research focuses on those in the earlier stages of illness, and does not investigate how coping strategies evolve [12, 28–30]. If we know that emotion and problem focused coping and self-care strategies can influence the negative consequences of the experience of advanced cancer[20], it is vital that we explore these issues in more detail to understand how to enable their support and use[31]. What is needed is further research on the dynamics of wellbeing and coping, particularly within patient/carer dyads, to expand understanding of the way in which psychosocial support needs might be addressed[32]. By exploring individual patient and carer perspectives on the development, use and appraisal of specific coping strategies the study addresses this gap in knowledge.

## Materials and Methods

### Research questions

This study was guided by four research questions:

1. What do people do to cope well when living with advanced cancer?
2. Why and when do they perceive these coping strategies as effective?
3. How can health care professionals support effective coping strategies?
4. What would be useful to support self management and effective coping strategies as the first step in developing a patient and carer led intervention?

### Study design

The study used a longitudinal qualitative design: serial qualitative interviews[33] were conducted both with people with advanced cancer and their family or other informal carers. Qualitative research enables collection of rich data on experience and behaviour changes over time in response to multiple stimuli. The focus of these interviews was to understand how people cope well when living with advanced cancer, and changes in coping strategies over time. Focus groups towards the end of the study with people with advanced cancer and their family/informal carers explored findings from the interview phase to determine how effective coping strategies could be best supported. The protocol for the study has been published[34]. COREQ guidelines were used to structure reporting[35].

The conceptual basis for the study is the process of adaptive coping, a model which highlights how the development of ‘positive emotion’ is not only the result of coping strategies but also simultaneously underpins ongoing positive coping[36, 37]. This model guided the study throughout, in particular the conceptualization of the interview topic guide and interpretation of data.

### Sampling

People with advanced cancer were eligible to be recruited to the study if they met the following inclusion criteria:

Patient inclusion criteria:

1. Adults > 18years. No upper age limit.
2. With advanced cancer, defined as those with metastatic disease at diagnosis, and/or where disease is progressing following treatment (local or metastatic spread) and/or where the prognosis is estimated to be less than a year[38].
3. Those whom their health care professionals judge have capacity to give informed consent to participate in the research.

With the permission of the person with advanced cancer, their family or other informal carers were also invited to participate if they met the following inclusion criteria:

1. Adults > 18 years. No upper age limit
2. An informal carer for a patient recruited to the study and identified by the patient as the person they get most support from, not necessarily a family member.

The anticipated sample size was 30 people with advanced cancer, and 30 of their family or informal carers. This is a relatively high sample size for qualitative research, but as these were people with advanced cancer we anticipated a high attrition rate due to illness or death over the course of the study. We therefore planned to over-sample at time point one to enable an adequate sample size at time point two.

### Recruitment

People with advanced cancer were recruited from outpatient clinics (lung, breast, prostate, colorectal and palliative care) within two tertiary cancer centres in the North West of England. This study was adopted onto the NCRN study portfolio, enabling the use of research nurses within these clinics to identify (from clinic records) and approach those who met the inclusion criteria. Regular monitoring of the study population focused on ensuring that potential participants were invited to participate from a range of tumour groups, with variability in age and gender where appropriate.

Patients were identified and approached between May 2012 and April 2013, with recruitment ceasing when data saturation was reached (for the first round of interviews). Patients were given two participant information packs during a routine clinic visit, and asked to give one, designated, pack to ‘the person who gave them most support’ (informal/family carer for the purpose of the study). If interested in study participation they informed the study team via a Freepost response form which enabled people with advanced cancer and their carers to make independent decisions on participation. Responding participants were telephoned to schedule the first interview, with arrangements for the second interview completed at the close of the first visit. Second interviews were scheduled to respond to any anticipated disease progression (e.g. participants with lung cancer were offered interviews towards the minimum protocol timescale of 4 weeks), and life events and clinical milestones such as consultations or diagnostic results identified by participants.

Full written consent was taken separately from each participant on each visit immediately before the start of the interview, with process consent procedures followed throughout the study to respond to any issues of distress or discomfort. Participants could withdraw from the study at any time.

Towards the end of the study a number of those who had participated in individual interviews were invited to take part in focus groups. Information about this had been given at the time of initial recruitment. Health status was checked with the recruitment site prior to letters of invitation sent to those considered clinically able to participate in June 2013. Carer participants associated with patients who were too ill or who had died during the study were excluded from invitation.

### Data collection

Data were collected through serial qualitative interviews and focus groups.

#### Interviews

Interviews aimed to collect rich and detailed data examining the coping strategies people employed, why strategies worked, for whom and under what circumstances. Separate interviews with people with advanced cancer and their nominated carer enabled exploration of both individual and shared coping strategies. Conducting serial interviews enabled exploration of how strategies and perceptions may evolve over time, and coping as a process[33, 39].

The initial interview topic guide developed iteratively through the study (see Table 1 for final version of topic guide), but individual interviews were participant led, enabling a flexible response to emerging issues. Interviewees knew the focus was on coping with advanced cancer, as this was clear in the information sheet and consent forms. Interviews were conducted in a setting of the participants choice (all interviews were conducted in home settings), and were digitally audio recorded to enable verbatim transcription. Mean initial carer interview length was 62 minutes (range 35–92 minutes), and mean initial patient interview length was 64 minutes (range 42–106 minutes). Interviews were conducted by DR (an experienced qualitative researcher with particular expertise in gerontological research) and listened to/read by CW (an experienced qualitative research with particular expertise in palliative care research). After each interview reflective field notes documented observations to inform analysis.

**Table 1:** Final version of interview topic guide.

General probes: How did that make you feel? How did you deal with that? How do you think you will manage that? What do you think will happen?
Prognosis—What have you been told about your illness? Immediate reactions Perception of illness—What did you think that would mean? Current situation—Do you know how it developed? Information Access—What have you found out about your illness and how? Wellbeing—What makes you feel you’ve had a good day? Coping—How do you manage the difficult aspects of being ill? Aims—Have you chosen to do any new things since being ill? Changes—Is there anything you do differently than before, better or worse? Significant events— Perception of illness—Has how you feel about being ill changed since you first found out? Anything else to add?

#### Focus groups

Qualitative focus groups with people with advanced cancer and their informal carers were conducted to debate and discuss the coping strategies identified in the interview phase of the study, consider prioritisation of strategies, and determine how people with advanced cancer could be enabled to develop effective coping strategies. Separate focus groups were held for those with advanced cancer and their informal carers, as it was not known if they may have separate views on interventions. Focus groups were conducted in both recruitment locations to facilitate participant access, with participants provided with refreshments and reimbursement of any out of pocket expenses. Focus groups were facilitated by CW and DR, with assistance from moderators who took notes on a flip chart and provided a verbal summary at the end of each focus group. Focus group discussions were digitally audio recorded and transcribed verbatim. Focus group discussions lasted a mean of 74 minutes.

Stimulus material for focus groups was derived from theanalysis of interview data, with a focus on themes with potential for intervention development, guided by our research questions [40]. Participants were individually provided with stimulus cards based on themes in a random order at focus group commencement and asked to individually consider the issues on each card, put them in personal order of priority, and then place them face down in one of three areas labelled ‘most important’, ‘useful’ and ‘nice to have’. The focus group facilitator collated the cards and facilitated discussion to reflect themes in descending order of priority.

Both individual interviews and focus group interviews could potentially cause distress, and a clear distress protocol was in place, approved by the research ethics committee.

### Data analysis

#### Interviews

Constant comparison techniques were used in the initial fine grained analysis of interview data[41]. Interviews were coded (using the software QSR NVivo 10^™^) independently by DR and CW, with regular discussions to confirm interpretation and revise the emergent coding structure. Codes were derived *a priori* (from topic guide) and also iteratively from first and second interviews of people with advanced cancer and their informal carers. To facilitate the visibility of longitudinal factors in analysis data codes were colour coded to flag the point at which they emerged in the data collection process.

The matrix coding query facility within NVivo 10^™^ was then used to compare and contrast inter and intra group coding for people with advanced cancer, informal carers and patient/carer dyads at and between first and second time points. Each interview transcript was created as a case node and multiple matrices produced to cross-reference individual, paired or multiple case nodes against selected codes within each matrix. This exploration of different aspects of the data from the qualitative serial interviews enabled identification of common factors within interview narratives and highlighted differences between patients, between carers, within dyads and over time. All interviews contributed to the analysis, even where a second interview was not possible due to deterioration or death, although for some dyads change over time could therefore not be analysed. However, participants were all at different timepoints and disease stages from diagnosis of advanced cancer, and hence their data important whenever it was collected.

At intervals the coding structure and anonymised data extracts were discussed and revised with the research advisory group to facilitate a range of clinical, academic and lay perspectives in the analytical process.

#### Focus groups

Analysis of focus group data involved both the use of frequency counts in the collation of stimulus material across focus groups and thematic analysis of the focus group participant narrative. QSR NVivo 10^™^ was used to collate views on the stimulus material, and transcriptions coded with a focus on identification of how high priority topics might be translated into a future intervention.

### Ethics and governance approvals

NHS Research Ethics committee approval was given by National Research Ethics Service North West—Cheshire (11/NW/0739) and governance approvals given by both tertiary cancer centres. No treatment or other relationship existed between patients and key contacts or any member of the research team. No personal or clinical details were provided by the recruitment sites with the exception of patient name, gender, age and tumour type. Written consent was given by all participants. Direct telephone or face-to-face contact with responding participants and access to raw study data was limited to DR and CW.

### Data management

Data were collected on encrypted digital audio devices, and downloaded as soon as possible to a secure password protected server at the host University. Files were transferred to a transcription service, with confidentiality agreement in place, via a secure password protected file transfer protocol. Audio files, transcriptions and NVivo files are stored on the secure server for 10 years.

## Results

### Participants

Fifty participants were interviewed (people with cancer n = 26, informal carers n = 24) for the study between July 2012 and May 2013, resulting in 86 individual interviews (people with cancer n = 45 (first interview n = 26, second n = 19), informal carers n = 41 (first interview n = 24, second interview n = 17) at two time points. Not all participants were able to complete two interviews due to illness progression or death. The time point for the second interviews ranged from 4 to 12 weeks after the first interview. Four focus groups were conducted in July 2013 with 8 people with cancer and 8 informal carers.

Participants are identified in the data extracts below by alphanumeric code where P = patient participant, C = carer participant, IV 1 or 2 = whether first or second interview and FG 1 or 2 = focus group by recruitment site (P or C number for focus groups = speaker order and does not relate to study ID).

Participant summary details are given in Table 2.

**Table 2:** Participant demographic information.

	Patient participant	Carer participant
Number	N = 26*	N = 24*
Gender	M = 17 F = 9	M = 6 F = 18
Age	Age range 32—82 (Mean 56.9)	Age range 28—74 (Mean 52.5)
Relationship to patient participant		Spouse = 17 Child = 4 Parent = 2 Sibling = 1
Recruited from tumour group clinic	Breast = 4 Prostate = 3 Lung = 8 Colorectal = 9 Other (via palliative care clinic) = 2	
Occupation	Retired = 12 Working = 13 Home maker = 1	Retired = 6 Working = 12 Home maker = 6

*1 carer participated without the patient participant taking part, 3 patients participated without nominating a carer. All other patients and carers were dyads.

### Coping strategies

Coping with advanced cancer appears to result from small scale changes in ‘everyday’ behaviours or perceptions, and is a complex and dynamic process. The process of coping reflects past coping capacity based on life history and/or inherent psychological traits but also encompasses responses to the fluctuating impact of current illness and life events. Coping does not appear directly related to disease type, disease trajectory, age, gender or demographic factors. Data are presented first on development of coping strategies, second on types of strategies used, and finally on how to promote the development of effective coping strategies.

#### Developing coping strategies: Coping as a continual and changing process

The ability to cope with living with advanced cancer as a patient or family carer fluctuates, with individuals combining perceptions of illness, responses to life events and inherent psychological traits to identify, develop and use effective coping strategies:

*…I don’t do low. There’s no point. I can’t see the point of it. Negativity does not help …. I think it’s very important. It’s not a case of maintaining it. It just happens. There’s no effort required. I’m just this way. …. It’s just part of me. I am me*.

(P23, IV1A)

There does not appear to be a linear development of the ability to cope, nor necessarily long term maintenance of coping at a particular level. Participants describe positive and negative changes to coping resulting from an evolving understanding of their disease and its impact on everyday life and future plans:

*You try to keep things normal, but it isn't normal anymore … It's as normal as you can make things, but it isn't normal…I don't mean everything is an effort but there is a lot more thought having to be put into everything*.

(C03, IV1)

Wellbeing fluctuates over time and in response to life events beyond association with disease trajectory or treatment cycles and individuals deploy a range of coping strategies dependent on purpose, resources and social role:

*… once I’ve got [my hair back] I can start going out with [husband] a bit more and… when I go out for a meal with them all [whole family], like, I’d go mad and put my wig on and that, but it’s not comfortable…. I love it when that door is shut and I can just lie on there with nothing on my head*.

(P21, IV2)

For individuals, the movement between perceptions of negative and positive coping has an identifiable pattern. Both patients’ and carers’ narratives indicate identifiable ‘low points’ such as diagnosis or adverse test results from which there is a need to move toward a ‘high point’ of positive perception but that neither extreme represents a permanent change:

When I actually had to go for the MRI scan or the CT scan, I would be very anxious getting those results because I don’t want to know what they’re going to tell me, and I will find that… What they’ve got to tell me, yes?

(P24, IV1)

Individuals commonly refer to ‘*going round and round*’ to describe revisiting earlier perceptions and coping less well before finding ways of coping more effectively and regaining wellbeing as a result. Peaks and troughs of wellbeing appear transient although the time period of change is variable.

*So yeah it was quite shocking when I was first told. And then you sort of get your head round things and say okay let’s see how we get on*.

(P04, IV1)

The pattern repeats over time, ‘looping’ as individuals not only move between positive and negative emotions but revert to an earlier point as they take time to reflect and recover or lose wellbeing.

#### Developing coping strategies: Evolving strategies and their influence on perception of coping

The ability to cope, and the sense of wellbeing that effective coping engenders, fluctuates. This may be related to the different coping strategies that people use. There appears, for example, a distinct contrast between coping strategies employed immediately after diagnosis which may reflect reactions to ‘loss’ and those used over time which tend to reflect individualised, pragmatic objectives.

*But I think when I was first told I thought, well, with the treatment that would be the end of it. And then everything’s…well, I put in for my blue badge and I got the diagnosis written down off the doctor, and I think it sunk in more than when I’d seen it down in black and white: terminally ill and all the rest of it. And I thought, they’re talking about somebody else*.

(P21, IV2)

Both within and between interviews participants clearly indicated that their initial response to diagnosis and their ways of ‘*dealing with it’* had changed over time. They commonly described emotions and perceptions on receiving the diagnosis as ‘*frozen*’ or that they ‘*couldn’t take it in’* but all were subsequently able to recognise ‘*that day’* as an event, reflect upon it and to recognise that perception had shifted.

Initial struggles to cope with the diagnosis were embedded in stories of shock, dismay, disbelief, fear of the unknown and grief which had, by the time of the interview, been replaced by presentations of acceptance and pragmatic descriptions of coping although the level of acceptance and recognition of coping strategies were usually greater when more time had elapsed since diagnosis. The individual and intra-dyadic narratives often described early reactions as ‘*not coping’* and continued by expressing participants’ recognition of the need to regain some degree of wellbeing.

Do we, you know, wallow in our pity and have what time we’ve got together, you know, we are not going to enjoy him, we don't want to remember it because it's that down? or do we make the best of everything and carry on as much as you possibly can, deal with it, being normal as normal as it is to get us through it?

(C03, IV1)

Coping in order to maintain, develop or regain wellbeing became the outcome of using a ‘toolkit’ of strategies from which individuals choose those which resonate most fully with their perception of the current situation, aspects of life which pre-dated the diagnosis of advanced, incurable cancer and their sense of self-identity. This ability to develop coping strategies over time appears to be an important component in achieving wellbeing. ‘Evolution’ in this context represents not only the process of collecting and building a ‘toolkit’ of strategies but also the ways in which such strategies may change as disease progresses or illness experience is gained.

### Types of coping strategies

Participants described four broad categories of coping strategies that they had developed and deployed: everyday pragmatism; self-awareness; relying on others and communication.

#### Everyday pragmatism

Within the broad theme of ‘everyday pragmatism’ three interlinked strategies were identified: being realistic, changing priorities, and a focus on the everyday. Many participants concluded that it was necessary to ‘be realistic’ and spoke of the practical ways which they engaged with aspects of living with advanced cancer. ‘Being realistic’ was defined as both accepting the presence of disease and engaging with the symptoms and realities associated with a prognosis of incurable disease and reduced life expectancy:

*…I’ve just taken each symptom as it’s arisen. I’ve had loads of extra drugs to take for each side effect [and] learnt that if you need them take them and don’t be silly about things. [I try to] get moving and sorted, even if I’ve had to rest later on and I eat when I can*.

(P26, IV2)

Participants identified that ‘being realistic’ was an important factor in maintaining wellbeing as it prevented or reduced the frustrations and disappointments of unachievable expectations. In this context, expectations and priorities ranged from the minutiae of everyday life based on a pre-diagnosis ‘normality’ such as eating particular foods or having meals at habitual times to more aspirational targets such as holidays abroad.

*I’ve stocked up on lots of food that I know I normally like…it’s been sunny, it’s been bright. There’s been the French open tennis on the telly which I’ve managed to watch for half an hour at a time and then crash out…*.

(P26, IV2)

Individuals’ depth of reflection and the complexity of their lives pre- and post-diagnosis differed but all had reassessed short and longer term priorities in order to *‘live around’* the various impacts of illness. Recognising this as a necessary perspective was present in all the narratives and appeared to be based on the belief that cause was less important than management so participants commonly refocused attention on the sometimes small but essential details of their current everyday normality:

if I’ve got a pain free day, if I’ve had a nice walk or I’ve been line dancing, then I’ve had a great day, …It’s anything I think, where you feel normal again, because most of the time I don’t feel normal at all. …

(P01, IV1)

The identifiable impacts of disease and treatment were thus conflated and integrated into daily life to make managing and ‘living around’ symptoms, side effects and treatment schedules easier. Nevertheless, patients’ ability to take part in ‘normal’ activities was often severely curtailed, particularly by nausea and fatigue, which had a knock-on effect on carers’ wellbeing with the result that coping strategies adopted by both patients and carers were affected by severity of symptoms and change over the course of the treatment cycle.

In ‘being realistic’ participants often remained reluctant to make immediate plans for future events and demonstrated the desire and ability to compartmentalise, especially in coping with fears for the future. These fears centred on: disease progression resulting in physical deterioration; individuals’ capacity for delivering/accepting care; support from services; arrangements for end of life; and effects of bereavement:

*I know there’s going to be worse times to come in the future. I know that but I won’t be down about it because there’s nothing I can do and it’s best to get on with it, you know, as much as you can*.

(P03, IV1)

Strategies also reflected participants’ cultural and philosophical values through references to ‘*just having to get on with it*’ or ‘*the way things are’* alongside their sense of ‘self’ as ‘*the sort of person who…*’. There was a sense of both having to deal with the ‘everyday’, but also that such activities and tasks enabled ‘getting on with it’:

*What helps me is me grandchildren… Maybe it’s because I’m little and the kids think I’m the same size as them! … I can play with them now but not the same game as I used to; but it’s just carrying on. It’s not feeling sorry for yourself. It’s just getting on with it, and that’s the best way I look at it*.

(P03, IV1)

#### Self awareness

Participants described a self-awareness of coping by strategies such as feeling good through planned or pleasant activities, switching off, or creating ‘good days’ and awareness of when good days might be possible.

Participants made conscious efforts to ‘feel good’ by acknowledging challenges such as poor appetite and fatigue, and so finding ways of enjoying food and/or getting enough rest was important both physically and psychologically. Carers often instigated these interventions, but patients also described these strategies:

*And food-wise as well …I don’t even like mushrooms and we eat mushrooms all the time now … there’s something in them that obviously my body needs …. I don’t really like mushrooms but they need to be there. And that’s what I do*.

(P23, IV1)

Embracing ‘treats’ and varying degrees or forms of ‘self-indulgence’ emerged as an important mechanism in developing the ability to cope and maintain a sense of wellbeing at a time of great stress. Receiving a prognosis which changed an individual’s or a dyad’s perspective on both present and future time acted as a strong catalyst in shaping their day to day behaviours. Without exception, all participants spoke of ways in which they ‘treated’ themselves or those they cared for either materially or intangibly. For some, this took the form of increasing the frequency of activities that would usually be regarded as luxuries such as a ‘*going out for a nice lunch*’ or exchanging a small gift aimed at pleasing the recipient while for others it comprised behaviours which were essentially ‘normal’ actions but simultaneously demonstrated thoughtfulness which was emotionally valuable.

*I think I’ve tried to be a bit more attentive, caring, well I’ve always been caring, but you know, yes attentive I think is the word, just holding hands and just yes*.

(C01, IV1)

Participants also acknowledged that they enjoyed activities which enabled them to ‘switch off’ from considerations of their diagnosis and prognosis, often through participation in everyday activities which others thought they should be protected from:

*But he thinks it’s terrible because [son’s partner] comes with [disabled grandson], and she’s here a lot, and he thinks it’s wearing me down. But it’s nice, like a breath of fresh air. And it takes your mind off what’s going on as well*.

(P21, IV2)

Such treats or everyday activities were not always possible and participants differentiated between days when they felt physically able to undertake tasks or activities and those days when they felt unwell and needed to rest. On the days where greater physical activity was possible, treats were often external to the home and involved activities which had been enjoyed pre-diagnosis such as shopping, visiting friends or playing with grandchildren. Activities once considered normal and expected were now re-categorised as ‘treats’ even where they were constrained to some degree by generally lower energy levels. When describing days where symptoms and side-effects were problematic the impact of fatigue was most commonly mentioned. For most people, the notion of staying in bed or sitting down reading for prolonged periods during a ‘working day’ was not only unwelcome but unfamiliar as time to ‘indulge’ had previously only been available once work tasks were completed. In accepting that managing symptom burden required acceptance of limits the inclusion of physically undemanding activities such as reading was therefore often described as a ‘treat’.

*I just do somet*h*ing that would please me*, *maybe doing a puzzle*, *because I quite like doing puzzles*.

(P01, IV1)

Patient participants also spoke of ‘wasted days’ and ‘lazy days’. They defined these as relating to the days when they were physically able but did not follow through on tasks or activities (wasted days) and those days when symptoms or side effects meant that they had to prioritise rest over activity (lazy days). The reasons given for differentiating in this way were that on the ‘wasted’ days the ‘ineffective’ use of time produced feelings of regret as ‘time’ in the sense of life expectancy was limited. For ‘lazy’ days there was little regret expressed as the time spent resting was perceived as necessary and legitimate:

*A wasted day is when you can do something if you wanted to but you choose not to. I could cut the grass or go for a walk or go for a drink but I choose to (rest… and) when I think I wasted yesterday, that normally gives me a gee up …. a lazy day is when I just can’t be bothered with anything or anybody (because I feel unwell)*.

(P04, IV1)

Both patients and carers spoke of ‘treats’ but not always in the same way which was indicative of the relationship within the dyad. Whilst all participants appreciated the value of a ‘treat’ in ‘lifting spirits’ it was not always a ‘treat’ for the person speaking. For some, the ‘treat’ was for the other in the dyad and generated feelings of wellbeing either from the act of giving or from receiving something, particularly if unexpected. The ‘giving’ of ‘treats’ was most commonly enacted by carers, particularly where the patient was feeling unwell or anticipating a difficult event such as a clinic visit. Carers across the sample described how they would ensure that the patient was psychologically supported by them as much as possible by planning ‘treats’ to coincide with difficult times.

*…if we get to [clinic] early enough … [there’s a nice pub and] on a day like today you can sit out and see [the marshlands], it’s lovely … or we go and see a little bit of different places when we go [to clinic] and make it a bit more interesting*.

(C01, IV1)

Participants discussed similar approaches to coping and maintaining wellbeing, despite different resources being available to them. This is particularly visible in the ‘treats’ which emerged as such an important element of the narratives but where greater social capital and material resources (including accessible transport and higher disposable income) increased the range and ease of access to options. It is a differential which applied not only to aspirations such as special trips or events but also to managing daily life. As a result, participants developed strategies to include low and non-cost ‘treats’ even where material resources were very limited. The changed everyday reality which results from living with advanced cancer also links not only to what people learn but also how, when, where and from whom.

#### Relying on others

Participants spoke of a reliance on others in enabling coping, through having people to turn to for emotional, practical and social support and through a reliance on and trust in professional staff. Many participants spoke of everyday support they received in meeting the additional demands that their illness and its treatment engendered. People were aware this was a reliance that may not previously have existed and of the balance required of receiving required support but not over burdening people:

*Somebody’s driving me [to clinic appointments], at the moment…. so I go all the way round my friends, they’re all doing one or two, so it’s not one person doing it, because it’s unfair, that*.

(P02, IV1)

However, accepting the need for support could be challenging for some, and also recognising that carers too benefitted emotionally from the provision of support. For some this was an adjustment which had taken time, moving from a ‘normal’ sense of independence to the recognition of the benefits of sharing illness burdens and the advantages this could bring to both:

He [husband] makes sure it all happens as it's meant to, yeah…Sometimes I think he's mollycoddling me a bit, but then he wants to do it, so I let him now. I don't argue with him now, I just let him do it…

(P20, IV1)

In addition, it was apparent that while people didn’t always ask for support they took time to recognise the importance of support offered by other people, and were grateful when others recognised their need:

*I just had the one, [when] I looked it up on the net, and it just happened that my sister called on the same day and so within about half an hour she was around here with chocolate, ginger beer and…I think she had…non-alcoholic ginger beer, and something else but she came with a bunch of stuff and sat with me for a while*.

(P23, IV1)

Family carers also spoke of the enabling features of both sharing the practical burden of caring and how this relieved their own concerns when such support was offered by those who were trusted:

*I’m very grateful that she’s got a good circle of friends… I know full well if my mother says she’s going out with friend A from church then I’m quite happy with that… I can go and do something and not worry about how she is, where she is or is she planning on coming home this afternoon or is she getting the bus back*.

(C02, IV1)

Such reliance was not only a physical reliance on others, but also through acknowledging that other people provided much needed emotional support. For carers, seeking emotional support from others was seen as a way of enabling them to return refreshed to the demands of being a carer:

…when [husband] is down I have to leave the house go to my sisters and I'm heartbroken myself and she’ll cry with me and [we comfort each other] then I have to come back and try and pick us up … so you really sometimes you force yourself to stay up there for the other person don't you?

(C03, IV1)

Participants also spoke of the support given and received at the recognised ‘stress points’ of consultations where participants recognised concerns about receiving information about disease progression or suggested further treatments:

*She likes having me [at the consultations] obviously, she likes the support, and that’s what I give as best I can without treading on her toes. But if she says I don’t want this then fine, who am I to say otherwise. I just respect that*.

(C02, IV1)

Nevertheless, people spoke about the importance of ‘managing’ who gave support and in what manner. It was recognised that the strengths and limitations of different supporters required appraisal, and in particular those who supported them in such stressful consultations needed clear guidance on what was wanted:

*but I have told her [daughter], I said, don’t be asking too much, I don’t want to hear it…I have got a husband but he works and…I shouldn’t say he’s not supportive but he can’t cope with anything that’s not very nice…I wouldn’t let him go to an appointment with me or anywhere; I’d feel that he can’t cope with things*.

(P24, IV1)

Patient and carer participants also spoke about the importance of being able to put trust in the professionals providing care. Trust wasn’t automatic, but had to be earned through their experience of working with a particular professional, group of professionals or organisation who demonstrated traits which engendered confidence:

*…whenever [the nurses and the oncologist and the surgeon] see me they say, oh, this is really good, this is good news, you’re doing well, this is good, I think that reassurance, which I know they wouldn’t just give for the sake of giving it, it’s making me feel very positive, yes. And I know they’re just not saying it because they couldn’t, they wouldn’t…or they’d just say nothing basically, yes*.

(P24, IV1)

#### Communication

Communication issues appeared vital both as a coping strategy in and of itself and in facilitating or enhancing other strategies. Participants talked about working with professionals, getting information, learning from or responding to others and communicating differently.

Participants required differing levels, amounts and timing of information to facilitate their coping, but this wasn’t always matched with the information they received:

I like to know exactly where I’m at and I like to know all the information. … because then I can compartmentalise and I can cope with it and I can deal with it, and my oncologist isn’t necessarily the easiest to squeeze information from because I think a lot of people are scared of knowing the full truth

(P01, IV1)

There was also a differentiation between the information people wanted to receive and the information they were prepared to communicate to others. Whilst complete openness was felt to be helpful with some members of a supportive circle, this didn’t necessarily mean that level of disclosure was felt to be helpful across the board:

*… I don’t think people really want to hear all the bad stuff. …I’ll talk to my husband and tell him more things, and perhaps my best friend and my daughter. But I think I try and make people feel that I’m okay*,

(P23, IV1)

This differential approach to communication mirrors the desire to ‘feel normal’ and to participate in conversations and activities which mirror, albeit sometimes in an amended way, the way that life was lived before the diagnosis of advanced cancer.

In developing coping strategies participants differentiated between knowledge gained from personal experience, health professionals, social contacts and other patients/carers. For some, the knowledge arising from personal experience was translated from other situations and supplemented by experiential knowledge within their own illness experience. For others, there was little history of similarly stressful, uncertain or difficult situations and knowledge was then gained from either current personal experience, health professionals or health literature provided through the clinic or other sources such as the internet. This latter source of information was of variable quality and utility, was described as ‘*useless*’ ‘*not very helpful’* and even ‘*frightening*’ to the extent that few participants indicated they found such information useful either initially or as an ongoing source of support.

*No, I haven't looked on the Internet or nothing, because somebody said to me it's the worst thing you can do, you'll find out things that you don't want to find out. And I just haven't bothered. I'm not an Internet person really*.

(P21, IV1)

Participants all indicated that the clinical teams at their respective clinics were very knowledgeable and approachable but emphasised that the knowledge garnered from peers was usually different in nature to that acquired from health professionals. Interactions in clinic waiting rooms or during treatment delivery therefore most often revolved around individuals’ perceptions of their current situation and focussed on the everyday aspects of living with advanced cancer rather than specific diagnostic or treatment information.

*…[it] was really nice to see that there were other people [like me] and they were coping… It was just really nice to talk to them and hear how other people manage their days really, and how they got through it, and you sort of compare yourself to them, don’t you, and you think, “Oh I could do that”, and “That’s a good idea”, or “I don’t do that”*.

(P01, IV1)

However, although most participants reported helpful conversations with other patients/carers during clinic visits many of the strategies that participants employed had emerged from ‘learning the hard way’. They thus highlighted how helpful it was to have contact with someone who had existing experience of dealing with aspects of treatment or managing fluctuating energy across the treatment cycle.

### Prioritising and promoting the development of effective coping strategies

Focus groups of participating patients and carers were specifically asked to prioritise the coping strategies which emerged from analysis of individual interviews, and then to discuss how such strategies could be enabled for people. Table 3 displays the information which was displayed on cards for them (topic and descriptor) and how many participants indicated the topic was most or least important or ‘good to have’.

**Table 3:** Results from card sorting exercise in focus groups.

Topic	Descriptor	Number of patients identifying as ‘most important’	Number of carers identifying as ‘most important’	Number of patients identifying as ‘good to have’	Number of carers identifying as ‘good to have’	Number of patients identifying as ‘least important’	Number of carers identifying as ‘least important’
**Everyday pragmatism**							
**Being realistic**	Accepting need for change, being ‘sensible’, balancing what gets done against available energy	4	3	1	2	1	2
**Changing Priorities**	Revaluing what is important within new limits created by illness	1	5	4	2	1	0
**Everyday things**	Managing the things that make up ‘normality’ and everyday tasks/activities	1	2	4	4	0	1
**Self awareness**							
**Switching Off**	Finding ways to 'switch off' thinking about illness, impact, prognosis	3	4	2	3	1	0
**A Good Day**	Reflecting on the things that make a day feel like a ‘good day’.	4	3	0	3	2	1
**Being self aware**	Thinking about own strengths or weaknesses, especially where these help in coping with or without help from others	3	1	2	5	1	1
**Pleasing yourself**	Putting pleasure above duty	0	0	2	1	4	6
**Relying on others**							
**Professionals**	Being confident in, comfortable with and trusting of health professionals	3	7	1	0	2	0
**Having people to turn to**	Being able to confide, offload, share time without a focus on illness, rely on help when needed.	3	3	1	3	2	1
**Socialising**	Feeling able to socialise with existing friends/family or to meet new people	3	2	1	4	2	1
**Responding to others**	Being able to see other people’s behaviour as supportive (even where this may previously have been unwelcome).	0	2	5	1	1	4
**Communication**							
**Information**	Getting information in enough detail at the ‘right’ time	3	5	2	2	1	0
**Communicating differently**	Being able to talk or act differently to allow feelings/needs to be shared.	1	2	2	3	3	2

Numbers represent the numbers of topic cards placed in each of three piles (most important, good to have, least important) within the focus groups.

The area which more people, especially carers, indicated was ‘most important’ was having trust in professionals, showing their core importance at this time in people’s lives:

Well, I think a professional should tell you the truth. If you’ve not got that, I don’t think you’ve got anything, but they’re always there for you. I mean, when I come here they’re there 100 per cent, you know what I mean

(FG1, P1)

However this was tempered with an understanding that the onus could often be on the patient or their carer to extract information from professionals rather than it being provided automatically:

*And you have to ask them the right questions to get the answers out of them, whereas they should give you all of the answers straightaway. You have to have enough nous to ask the right questions to get through to them*.

(FG1, P3)

These issues were related to the next most prioritised aspect (again particularly by carers) as getting information in enough detail at the right time. The issues particularly prioritised by patient participants in the focus groups were being realistic and reflecting on things that make a ‘good day’:

P3: The more good days you have, the better…

P4: The better…

*P3: …you feel about yourself and what’s going on*.

*P2: Sometimes on a good day you don’t even think about what’s up with you*.

*P3: No*.

*P1: You don’t*.

*P2: Especially if you’ve got other people there, coming in and out*.

P3: But a good day can be anything. It doesn’t have to be anything special…

*P4: Getting out in the garden*.

P1: No you don’t have to…

P3: …it can just be sitting in the gazebo in the garden reading and just not having a day with any pain in my arm. That constitutes a good day. It doesn’t have to be anything special, really, just a simple thing but it is important…

(FG1)

As well as discussing the rationale for their prioritisation of the importance of different coping strategies, focus group participants also discussed how they would like to learn about developing such strategies. Both groups of patients independently identified the idea of a mentor as important:

*I really like the idea of having a mentor, perhaps someone who has survived cancer who knows, rather than a professional as it were having…you know, recruit from people who have experience, gone through exactly this*.

(FG1, P4)

It was clear that many wanted this mentoring to be a personal, individual experience rather than a support group or similar, although some were flexible about a group:

*P4: Yes, but people don’t necessarily want to go to a group*.

P3: No, they just want personal, somebody…

P4: If you’ve got someone coming and…

*P1: The personal touch*.

*P3: Yes*.

P4: …taking you out for a cup of tea, you know, taking you down the café and whatever and the garden centre or…

*P3: Discussing, yes, if you want to talk about anything they can do*.

(FG1)

They also wanted a mentor or buddy relationship to be proactive—offered to them—rather than something that they had to initiate or seek out:

*Because you’re all in the same boat. And so I think he has found it helpful meeting other people. …But I’m not sure that he would have sought that out himself*.

(FG4, C3)

They were equally clear about the limitations of other ways of sharing information such as written information, because leaflets or information on websites could be impersonal and ‘scary’:

*P3: You do need leaflets*.

P1: Yes, but not for scaring…

*P3: But not for personal conditions*.

*P4: No, I didn’t like the leaflets because I’ve got bowel cancer and when I looked at the bowel cancer for stage four, they had a whole bunch of case studies of people and all of them were terminal. I thought, you’ve got just include, one hopeful. I’m reading through this and I’m like, this is…*.

(FG3)

## Discussion

### Principal findings of the study

People with advanced cancer and their informal/family carers are able to develop coping strategies which often enable psychological wellbeing to be managed or regained. These strategies draw from those which people used before their diagnosis, but develop through responding the experience of living with advanced cancer. Important strategies are described including everyday pragmatism, self-awareness, reliance on others and communication. Whilst people with advanced cancer recognise the importance of information from, and a trusting relationship with, their health care professionals, they wanted to learn about developing effective coping strategies earlier in their disease trajectory from their peers.

### Strengths and weaknesses of the study

This study has a number of strengths: it is a large, longitudinal qualitative study, where detailed data analysis is possible through drawing from a rich data base of in-depth interviews pertinent to whether, how, and what coping strategies were developed. Participant recruitment was broad, with a range of ages, gender, living and working status, and cancer diagnoses represented in the sample. A flexible interview protocol enabled us to work with participants to capture data at meaningful time points without the attrition common in some studies at the end of life[42, 43]. The study demonstrates again that it is possible to engage people who are nearing the end of their lives in research, and that they are willing to participate for the benefit of others who develop advanced disease.

We were not however, able to capture the experiences of the very young (under 30) or very old (over 85) in this study as none in these categories were identified or recruited, and it may be that their coping strategies or ways that they wish to develop such strategies differ. In particular those participating in the focus groups to discuss ways of promoting effective coping strategies were primarily aged between 50 and 60, and they may have particular ways of promoting coping strategies which were over emphasised in the research. However age did not seem to be a factor except in the degree of ‘timeliness’ perceived regarding their terminal diagnosis. For day to day coping the mechanisms described were common across participants, irrespective of their age. We also recruited participants from regional cancer centre clinics, and it may be that people who attend such centres differ in some unknown ways to those who do not.

### What is already known and what this research adds

The design of the study provides valuable research and practice perspectives on coping with living with advanced cancer. Coping research is predominantly quantitative, based on measures of anxiety, stress or depression as markers of negative coping, quantifying the relationship between coping and health and wellbeing, and focused on the experience of those in earlier disease stages[12, 29, 30, 44–51]. Whilst such research is important in identifying the importance of coping, and its interrelationship as a concept with aspects of wellbeing, it does not provide a rich understanding of the reasons, processes or types of strategies that people use to deal with advanced cancer[31]. Such rich understandings can help provide the practical guidance that health care professionals and people with advanced cancer need to understand what are realistic and workable everyday pragmatic strategies to promote coping and wellbeing.

Research into coping and/or living with cancer which has taken a qualitative approach supports many of the main findings of this study: that positive experiences are important and strategies for living can be developed[52]; that whilst living with cancer is disruptive it is possible to make sense of the experience and ‘find a path’ which enables coping[53]; that a focus on life priorities and everyday tasks such as work or hobbies can be helpful[13]; the importance of ‘holding on to life’ by enjoying life in the present[14] that people use emotional and problem focused coping strategies, often focused on maintaining normality[19]; and that supportive relationships with and trust in clinicians are important[19, 54, 55]. The narratives commonly included expressions of ‘hope’ which is a factor considered by other work on coping with cancer [56]. However, we did not include ‘hope’ as a distinct coping strategy because it was frequently paired with ‘resignation’, and hence was contextualised as an aspect of everyday pragmatism to show how people were able to express both hope and resignation concurrently. This study extends this knowledge by confirming factors in a larger, longitudinal study with a population of those with advanced cancer and, through its explicit focus on unpicking and understanding pragmatic coping strategies, the basis of potential interventions to promote and support such effective strategies.

A clear message from this study is that whilst supportive relationships with, and trust in, healthcare professionals is important, participants wanted a different, peer delivered, style of intervention to promote effective coping strategies. Participants value and respect health professionals’ input but are commonly reluctant to raise the ‘small’ issues which underlie the concerns visible as anxiety in consultations. Peers are able to provide pragmatic experiential information communicated differently than health professionals. Importantly, although questions about clinical aspects may arise from peer communication, participants focus mainly on non-clinical information. Peer support and input therefore appears to be an extremely important factor in developing an optimum sense of wellbeing. Research shows that supportive relationships with social networks, and peers with cancer are important, but can be limited after treatment completion[53]. There are important differences in the content and style of peer interactions in comparison to those with health professionals[57], and so we expect peer mentoring to have different outcomes to professionally mediated interventions. Peer support is known in a number of different health fields such as infant nutrition[58], vascular disease[59], diabetes[60], and mental health[61]. Whilst peer support is used in those with cancer, there appear to be no studies of peer mentoring specifically for those with advanced cancer[62–64]. Most peer support is offered in groups[62, 63], with a developing focus on internet facilitation[65–68], there is no research evidence for individual peer support for those with advanced cancer, as proposed by participants in this research.

### Implications of the study

The results of this study are important as they demonstrate that people with advanced cancer do cope well, but that such coping strategies are unlikely to be promoted or discussed during their interactions with health care professionals. Recognising this indicates that a different approach to care is required to enable the speedy adoption of effective ‘everyday’ coping strategies, and that this may be a peer mediated approach. This is not reflected in the current models of psychological support provided by the NHS which focuses primarily on the level of expertise of the health care practitioner supporting the patient with psychological problems[69]. These approaches do not reflect increasing demands for participatory, community approaches to care based on social action models which are not necessarily professionally mediated[70]. Research is needed which explores how the potential of peer contact can be harnessed, and considering different models of peer support for those with advanced cancer.

## Conclusion

Our data show that psychological wellbeing for those with advanced cancer can be enhanced and regained through individual coping strategies. Providing people with advanced cancer and those supporting them with congruent information about using such strategies whilst living with advanced cancer is therefore fundamental. Health professionals are well placed to identify people with advanced cancer and carers who might benefit from access to such information about effective coping strategies. As a result, they have a key role in ensuring that patient-developed wellbeing is not undermined by focussing solely on clinical aspects in consultations. Services and individual health professionals need to recognise patients’ and carers’ desire for information beyond the clinical and to embrace the potential offered by informal, pragmatic input from patient/carer peers by establishing and signposting appropriate initiatives and opportunities.
